# Gonadoblastoma with Dysgerminoma Presenting as Virilizing Disorder in a Young Child with 46, XX Karyotype: A Case Report and Review of the Literature

**DOI:** 10.1155/2022/5666957

**Published:** 2022-05-23

**Authors:** Prathamesh Chandrapattan, Amitabh Jena, Rashmi Patnayak, Swayamsidha Mangaraj, Sujata Naik, Saroj Panda

**Affiliations:** ^1^Department of Surgical Oncology, IMS & SUM Medical College and Hospital, Bhubaneswar, Odisha, India; ^2^Department of Pathology, IMS and SUM Medical College and Hospital, Bhubaneswar, Odisha, India; ^3^Department of Endocrinology, IMS & SUM Medical College and Hospital, Bhubaneswar, Odisha, India; ^4^Department of Medical Oncology, IMS & SUM Medical College and Hospital, Bhubaneswar, Odisha, India

## Abstract

Gonadoblastoma is a neoplasm containing an intimate mixture of germ cells and elements resembling immature granulosa or Sertoli cells. It has been considered as in situ germ cell malignancy that can be associated with malignant components. The tumor has been reported to almost exclusively develop in various types of gonadal gene mutation syndromes, such as in pure or mixed gonadal dysgenesis and among females carrying Y chromosome material. However, it can be rarely present in normal women with 46, XX karyotype. Ovarian gonadoblastoma presenting with signs of contrasexual puberty in a young female child with normal 46, XX karyotype is an extremely rare clinical entity and seldom reported in the literature. We report a case of a nine-year-old girl child who presented with signs of virilization and contrasexual pubertal development. A detailed clinical evaluation along with supportive biochemical and radiological findings pointed to the presence of a virilizing ovarian tumor. The patient underwent right salpingo-oophorectomy, pelvic node dissection, and infracolic omentectomy. The excised tumor was confirmed to be gonadoblastoma which was overgrown by dysgerminoma on histopathological evaluation. The presence of associated malignant tumors (like dysgerminoma) should always be ruled out in cases of gonadoblastoma.

## 1. Introduction

Various inherited and acquired causes can lead to hyperandrogenism and its resultant manifestations in a female child. In young children, androgen-producing ovarian and adrenal tumors can lead to features of virilization in a relatively short span of time. Gonadoblastoma is a relatively rare ovarian tumor comprised of sex cord and primitive germ cell components. Though being benign themselves, they are frequently associated with invasive germ cell malignant tumors [1]. These tumors are frequently seen among individuals with 46, XY gonadal dysgenesis. However, they have been rarely described in individuals with normal 46, XX karyotype [1–6]. This presentation is even rarer in children less than ten years, and only one such case has been previously reported [5]. We present an interesting case of a young girl who presented with features of contrasexual pubertal development which was subsequently attributed to right ovarian gonadoblastoma with dysgerminoma. We describe her clinical presentation and management outcome along with a review of the literature describing cases of gonadoblastoma arising in individuals with normal 46, XX karyotype.

## 2. Case Report

**Figure 1:**
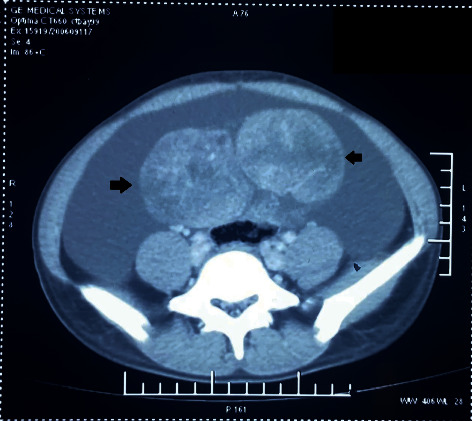
A large lobulated solid heterogeneously enhancing mass (solid black arrows) arising from the right ovary of size 11.9 cm × 6.5 cm × 9.4 cm with punctuate internal calcification and ascites.

**Figure 2:**
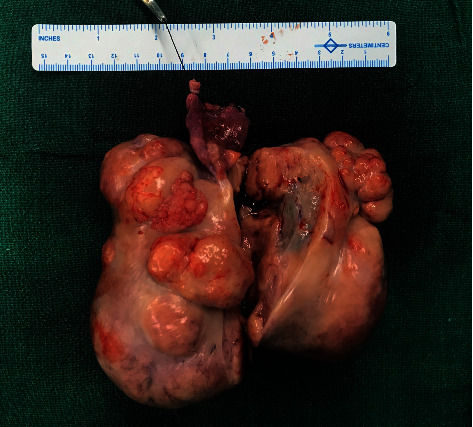
Resected specimen showing cut opened the right ovary with an attached fallopian tube. The right ovary measures around 15 cm × 10 cm with a bosselated outer surface. The ovary is entirely replaced by a solid mass which greyish-yellow with focal areas of myxoid changes and haemorrhage with an intact capsule. The fallopian tube appears normal.

**Figure 3:**
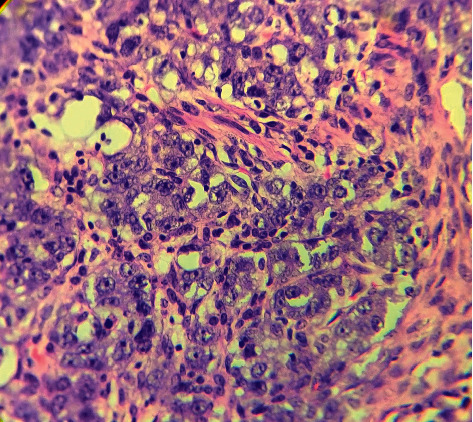
Histological findings showing nests of germ cells separated by fibrous septa infiltrated by lymphoid cells. Tumor cells are large, high N : C ratio with prominent nucleoli. (hematoxylin and eosin (H&E), 200x).

A nine-year-old girl presented for evaluation of progressively increased hair growth over androgen-dependent areas, deepening of voice, and abdominal distension. The patient had first episode of bleeding per vaginum at the age of 7 years and 6 months. Following it, she had an irregular menstrual bleeding pattern for the next five months. Subsequently, the bleeding episodes had ceased on its own without any drug intake, and she was amenorrhoeic for the last one year. However, no medical consultation or evaluation was done at that point of time for this precocious pubertal event. The parents had also noticed a significant height gain in comparison with her peers in the preceding one and half years. There was no history of any chronic drug intake or prior surgery. There was no history of a similar disorder in any family member. She was born out of a nonconsanguineous marriage and delivered at term by normal vaginal delivery. The perinatal period was uneventful. On clinical examination, the girl was thin built with muscular appearance. Her anthropometric parameters were as follows: height of 145 cm (>97^th^ percentile) and weight of 41 kg (90^th^–97^th^ percentile). She had evidence of virilization characterized by a significant degree of hirsutism (Ferriman–Gallaway score of 16/36), clitoromegaly (clitoral length 16 mm and clitoral index 56 mm^2^), and lack of breast development. No Turner syndrome stigmata were noted. Her vitals were within a normal range. On abdominal palpation, a mobile abdominal mass (around 10 cm × 10 cm) was noted in the right lower abdomen with the presence of shifting dullness and bulging flanks. Ultrasonography of the abdomen and pelvis revealed a large lobulated adnexal mass (around 11 cm × 6 cm × 9 cm) arising from the right ovary along with ascites. The left ovary was morphologically normal. A pubertal uterus (dimension of around 7.5 cm × 5 cm × 3.6 cm) with a normal endometrial echogenicity of 3 mm was also noted. Her bone age was advanced by two years in contrast to her chronological age. Contrast enhanced computed tomography revealed a large lobulated solid mass of size 11.9 cm × 6.5 cm × 9.4 cm arising from the right ovary, morphologically normal looking left ovary, and presence of gross ascites (Figure 1). The right ovarian mass was characterized by heterogeneous post-contrast enhancement and punctuate internal calcification along with multiple intratumor tortuous vascular channels (Figure 1). Serum lactate dehydrogenase (LDH) (361 U/L, normal: 140-280 U/L), serum beta human chorionic gonadotropin (hCG) (447.2 m·IU/ml, normal: <5.0 m·IU/ml), and CA-125 (56.93 U/ml, normal: <35 U/ml)) levels were elevated. Serum alpha-fetoprotein (AFP) level (4.2 ng/ml, normal: <5.8 ng/ml) was normal. Hormonal evaluation revealed significantly elevated levels of serum testosterone (12.51 ng/ml, normal: 0.06–0.52 ng/ml), low luteinizing hormone level (0.26 m·IU/ml, normal: >0.6 m·IU/ml for pubertal response), and slightly elevated estradiol level (30 pg/ml, normal: <20 pg/ml). Serum dehydroepiandrostenedione sulphate (DHEAS) levels were within a normal range. Karyotype analysis from peripheral blood sample revealed a normal female 46, XX pattern. Fluorescence in situ hybridization (FISH) from peripheral blood did not reveal any Y chromosome material. Based on the above clinical, biochemical, and supportive radiological findings, a diagnosis of virilizing ovarian tumor leading to contrasexual puberty was considered. Intraoperative findings revealed a 15 cm × 10 cm firm and lobulated mass arising from the right ovary whereas the left ovary appeared morphologically normal. The patient underwent right salpingo-oophorectomy, pelvic lymph node dissection, and infracolic omentectomy along with left ovarian biopsy (Figure 2). Around three liters of straw-coloured ascitic fluid was drained. The patient had an uneventful recovery in the postoperative period. Ascitic fluid cytology was negative for malignant cells. Histopathologic examination of excised tumor revealed greyish yellow tumor with focal areas of myxoid changes and haemorrhage with an intact capsule. The tumor cells were present in sheets and nests separated by fibrous septa with lymphoid infiltration. Occasional primordial follicles were also seen in between. The tumoral cells were large with prominent nucleoli and mitotic activity. At places, bizarre tumor cells with areas of calcification (suggestive of burned out gonadoblastoma) were seen. Regional lymph node assessment revealed reactive hyperplasia. Based on the above characteristic findings, a diagnosis of dysgerminoma associated with burned out gonadoblastoma was made (Figure 3). The left ovary biopsy sample revealed normal ovarian histology. Pathological staging was pT1aN0Mx. However, a cytogenetic study for assessing Y chromosome material from affected ovarian tumor tissue was not performed. The patient was started on chemotherapy comprising of bleomycin, etoposide, and cisplatin combination for four cycles. The virilization features decreased over the course of next three months with significant improvement in hirsutism, voice change, and other physical characteristics. The patient is on regular follow-up for the past one year with no evidence of tumor recurrence till now and menstrual cycles have not resumed. The hormonal evaluation revealed normalization of testosterone levels (0.2 ng/ml) and normal early pubertal gonadotropin levels.

## 3. Discussion

The diagnosis of virilization in a young girl can be clinically challenging at times. The causes include various inherited and acquired conditions such as disorders of sex development (DSD), virilizing ovarian tumors, adrenal tumors, and exogenous androgen exposure. DSD such as various forms of congenital adrenal hyperplasia (CAH), ovotesticular DSD, and aromatase deficiency can present with features of hyperandrogenism in females. They can be distinguished from other tumoral causes of virilization by the presence of genital ambiguity since birth, presence of associated findings (maternal virilization during pregnancy in case of aromatase deficiency, history of salt wasting in certain forms of CAH), usually slower rate of androgenization in contrast to virilizing tumors and characteristic hormonal profile. Similarly, ovarian and adrenal tumors are usually characterized by rapid progression and more severe virilization in affected individuals. The presence of cushingoid features in addition to signs of virilization may point towards the presence of underlying adrenocortical carcinoma. Elevated androgen levels (serum testosterone level of more than 2 ng/ml and serum dehydroepiandrostenedione sulphate level of more than 8 *μ*g/mL) are characteristic of virilizing ovarian and adrenal tumors, and these tumors may be identified by necessary dedicated imaging studies. The common age of presentation of gonadoblastomas is in the second and third decade [7]. The common clinical manifestations of these ovarian tumors include hirsutism, virilization, menstrual abnormalities, and abdominal pain/distension [7]. The characteristic features of virilization seen in gonadoblastomas are due to excessive production of testosterone by these tumors [1, 8]. On the other hand, ovarian dysgerminomas are usually hormonally inert. Rarely ovarian dysgerminoma can be associated with elevated estradiol levels due to the presence of syncytiotrophoblastic giant cells or due to malignant transformation [9]. Pure gonadoblastomas are usually small in size but may acquire large size due to invasive component overgrowth [8]. Around 40% or more cases of gonadoblastoma are bilateral [7, 10, 11]. The pathological hallmark of these tumors is the presence of sex cord and primitive germ cell components. The presence of calcification serves as an important diagnostic clue [2, 7, 8, 10].

It is customary to rule out the presence of invasive malignancy in every case of gonadoblastoma. This is due to the fact that malignant germ cell tumors are associated with 50–60% of gonadoblastomas. The most common malignancy is pure dysgerminoma, whereas other variants include immature teratoma, embryonal carcinoma, yolk sac tumor, and choriocarcinoma [3, 4, 7, 12]. Gonadoblastomas are found among 25–30% of patients with XY gonadal dysgenesis and among 15–20% of patients with 45, X/46, XY karyotype [2]. This finding underscores the importance of karyotype analysis. Normal 46, XX karyotype is observed in rare cases [1–6]. Hence, cytogenetic assessment of Y chromosome in blood/affected tissue is advocated. In our case, although we had conducted FISH analysis from peripheral blood, we could not do cytogenetic assessment of Y chromosome from affected tissue. Pure gonadoblastomas are benign in nature, and it has been reported that cases with dysgerminomas also have a favorable prognosis [3]. However, association of other tumor types such as the yolk sac tumor may have unfavorable prognosis [3, 13]. We have summarized relevant cases of patients having 46, XX karyotype and gonadoblastoma described in the literature in Table 1 [1, 3–6, 8, 10, 11, 14–17]. Our case is unique as similar presentation is extremely rarer in children less than ten years and only one such case has been reported earlier [5]. Cases of dysgerminoma presenting with precocious puberty in children have also been described rarely in children [9, 18]. The presentation of 46, XY complete gonadal dysgenesis as pubertal virilizing disorder in adolescent due to underlying virilizing ovarian tumor (presence of concomitant gonadoblastoma and dysgerminoma) has been well described in the literature [19].

Surgery remains the main modality of treatment. The extent of surgery includes oophorectomy accompanied by salpingectomy, hysterectomy, omentectomy, and lymph node dissection depending on the disease status [3, 8, 11]. Germ line and tumoral Y chromosome analysis are helpful in deciding regarding contralateral oophorectomy in young patients keeping in mind fertility issues. The coexistence of invasive malignancy in gonadoblastoma requires adjuvant chemotherapy [2, 9, 11].

## 4. Conclusion

The presence of virilizing features in a young girl should be thoroughly evaluated. Gonadoblastoma is a rare virilizing ovarian tumor that usually arises in dysgenetic gonads. Although frequently associated with presence of Y chromosome, these tumors can rarely be seen in individuals with normal 46, XX karyotype. The presence of concomitant malignancy associated with gonadoblastoma should be always ruled out due to important therapeutic and prognostic implications.

## Figures and Tables

**Table 1:** Summary of similar cases published in the literature with gonadoblastoma and normal 46, XX karyotype.

Author	Year	Clinical presentation	Age in years	Precocity	Karyotyping	Laterality	Management	Additional findings in histopathology (in addition to gonadoblastoma)	Adjuvant therapy
Erhan et al. [14]	1992	Abdominal mass	26	No	46, XX	Right	TAH + BSO	Dysgerminoma	Combination chemotherapy
Obata et al. [15]	1995	Abdominal pain	10	No	46, XX	Bilateral	B/L oophorectomy	Left with dysgerminoma, right with yolk sac tumour	Combination chemotherapy
Zhao et al. [16]	2000	Abdominal mass	27	No	46, XX	Unilateral	USO + chemotherapy + later TAH + USO + LND + omentectomy	Choriocarcinoma, embryonal carcinoma, yolk sac tumor, immature teratoma, and dysgerminoma	Chemotherapy
Erdemoglu & Ozen [17]	2007	Abdominal mass	19	No	46, XX	Unilateral	Unilateral oophorectomy	Endodermal sinus tumor	—
Gorosito et al. [10]	2010	Pregnancy with ovarian mass	17	No	46, XX	Left	Left oophorectomy	Dysgerminoma	Chemotherapy
Yilmaz et al. [11]	2010	Abdominal distention with mass	20	No	46, XX	Bilateral	BSO	Dysgerminoma	Chemotherapy (bleomycin, etoposide, and cisplatin) and radiation
Esin et al. [3]	2011	Irregular vaginal bleeding Pelvic pain	15	No	46, XX	Left	Left oophorectomy with right ovary wedge biopsy	Dysgerminoma	—
Kanagal et al. [8]	2013	Abdominal distention and mass	14	No	46, XX	Left	USO + cytoreductive surgery + right ovarian wedge biopsy	Mixed germ cells and sex cord cell derivatives	Combination chemotherapy
Kulkarni et al. [4]	2016	Abdominal pain	20	No	46, XX	Left	USO + omental biopsy	Dysgerminoma	—
McCuaig et al. [6]	2017	Oligomenorrhea and menorrhagia	20	No	46, XX	Left	USO	Dysgerminoma with syncytiotrophoblastic differentiation	Observation
Roth et al. [5]	2019	Abdominal pain and mass	9	No	46, XX	Right	USO	Malignant mixed germ cell tumour	Cisplatin based combination chemotherapy
Rafeey et al. [1]	2020	Abdominal pain and mass	10	No	46, XX	Bilateral	USO with cytoreduction with right ovarian biopsy, para-aortic LN sampling with Partial Omentectomy	Dysgerminoma	Chemotherapy (Bleomycin, Etoposide and Cisplatin)

BSO: bilateral salpingo-oophorectomy, LND: lymph node dissection, USO: unilateral salpingo-oophorectomy, and TAH: total abdominal hysterectomy.

## Data Availability

Data are available on reasonable request to the corresponding author.
